# ^18^F-FDG and ^68^ Ga-FAPI PET/CT for the evaluation of periprosthetic joint infection and aseptic loosening in rabbit models

**DOI:** 10.1186/s12891-022-05537-w

**Published:** 2022-06-20

**Authors:** Yiqun Wang, Yu Li, Liang Han, Jun Wang, Cong Zhang, Erpeng Qi, Dongyun Zhang, Xiaojun Zhang, Yong Huan, Jiahe Tian

**Affiliations:** 1 Department of Nuclear Medicine, The First Medical Centre, Chinese PLA General Hospital, 28 Fuxing Road, Beijing, People’s Republic of China; 2 State Key Laboratory of Nonlinear Mechanics (LNM), Institute of Mechanics, Chinese Academy of Sciences, Beijing, 100190 China; 3 School of Engineering Science, University of Chinese Academy of Sciences, Beijing, 100049 China; 4 Department of Pathology, China-Japan Union Hospital of Jilin University, Changchun, Jilin China; 5 Department of Interventional Ultrasound, The First Medical Centre, Chinese PLA General Hospital, Beijing, People’s Republic of China; 6 Beijing Key Laboratory of Engineered Construction and Mechanobiology, Institute of Mechanics, Chinese Academy of Sciences, Beijing, 100190 China

**Keywords:** Periprosthetic joint infection, Aseptic loosening, ^18^F-FDG, ^68^ Ga-FAPI, Rabbit model

## Abstract

**Purpose:**

We built a joint replacement loosening model based on the original rabbit model of infection and evaluated the performance characteristics of ^18^F-FDG and ^68^ Ga-FAPI in evaluating infection and loosening.

**Methods:**

After surgery, the rabbits were divided into four groups, with six individuals in the control group and 10 each in the aseptic loosening, *S. aureus* and *S. epidermidis* groups. PET/CT and serological examination were performed three times at two-week intervals. After the rabbits were euthanized, micro-CT, tissue pathology, pullout tests and scanning electron microscopy (SEM) were performed.

**Results:**

The pullout test and SEM showed the feasibility of the aseptic loosening model. ^18^F-FDG showed similar performance in the control and loosening groups. The SUVmax of the *S. aureus* group was consistently higher than that of the *S. epidermidis* group. As for ^68^ Ga-FAPI, the SUVmax of the control group was lowest in the second week and gradually increased over subsequent weeks. The SUVmax of the loosening group began to exceed that of the control group after the second week. The SUVmax of the *S. aureus* group in the second week was the lowest among the four groups and increased as the number of weeks increased. The pathology results showed concordance with the performance of PET/CT. Linear regressions between SUVmax and serology showed that ^18^F-FDG was positively correlated with CRP and IL-6, while ^68^ Ga-FAPI revealed negative correlations with CRP and IL-6 in the second week and positive correlations in the sixth week. In addition, the SUVmax and MT(target)V of both ^18^F-FDG and ^68^ Ga-FAPI were negatively correlated with bone volume/trabecular volume (TV) and bone surface area/TV.

**Conclusion:**

In this longitudinal observation, ^68^ Ga-FAPI showed greater sensitivity than ^18^F-FDG in detecting diseases, and ^68^ Ga-FAPI had no intestinal or muscular uptake. The MT(target)V of ^68^ Ga-FAPI was larger than that of ^18^F-FDG, which meant that ^68^ Ga-FAPI had the potential to define the scope of lesions more accurately. Finally, the SUVmax of ^68^ Ga-FAPI could not differentiate between loosening and infection; further study of the diagnostic criteria is warranted.

**Supplementary Information:**

The online version contains supplementary material available at 10.1186/s12891-022-05537-w.

## Introduction

With the ageing of the population comes a demand to maintain the quality of life of an increasing number of elderly people; thus, it is foreseeable that the number of joint replacements will increase continuously over the next few decades. It should be noted, however, that the number of complications is also likely to increase; among the complications of joint replacement, aseptic loosening is reported to be the most common, and periprosthetic joint infection (PJI) is the most devastating [1].

^68^ Ga-fibroblast activation protein (FAP) inhibitor (FAPI), as the most promising radiopharmaceutical to emerge in recent years, has been addressed by an increasing number of scholars in the field of bone inflammation [2–5].

FAP, a type II transmembrane protein of the serine endopeptidase gene family, is rarely expressed in normal mature tissues [6]. FAP is secreted mainly by activated fibroblasts, which are abundant in the human body and are involved in infection response, inflammation, tumours and immunity [7]. FAP is expressed when cells and tissues are under pressure but not when the internal environment is stable. Specific manifestations of infection, such as fibrosis, chronic granuloma, activated fibroblasts, and proliferation of small blood vessels, are also accompanied by FAP expression [8]. In tissue remodelling, rheumatoid arthritis and osteoarthritis, FAP is also expressed and increases with the severity of tissue inflammation and bone destruction [9].

The abovementioned results demonstrated the feasibility of infection imaging targeted at FAP, and we have previously reported that ^68^ Ga-FAPI exhibits entirely different performance from ^18^F-fludeoxyglucose (FDG) in an animal model of infection [10]. However, in that study, no aseptic loosening model was developed, and the distinction between PJI and loosening has been a major focus of research among joint surgeons.

We have already constructed a new model of infection, and in this report, we modified this animal model to mimic aseptic loosening. The aim of this study was to explore the performance characteristics of ^18^F-FDG and ^68^ Ga-FAPI in PJI and aseptic loosening models.

## Materials and methods

### Animals

This experiment was approved by the Laboratory Animal Center of Chinese People’s Liberation Army General Hospital (2020-X16-93) and performed in accordance with the ARRIVE guidelines. Thirty-six New Zealand White rabbits (purchased from Jinmuyang Co., Ltd, Beijing, China) were acclimated to a new environment for two weeks and reared in individual cages at 24–28 ℃, 50–60% humidity, and a 12:12-h light/dark cycle.

### Surgery

Briefly, after anaesthesia (a mixed solution of midazolam, xylazine hydrochloride and sterile saline, 0.3 ml/kg), shaving, disinfecting and draping, a medial incision was made on the left knee. The capsule was exposed and, self-locking screws with a diameter of 3 mm and a length of 20 mm (Biortho Medical Science and Technology Co., LTD., Jiangsu, China) were driven into pilot holes drilled in the femoral intercondylar fossa and anterior cruciate ligament footprint. It should be noted that for the control and infection groups, the drill diameter was 2.5 mm, and for the loosening group, the drill diameter was 3 mm to simulate the initial loosening. After suturing the capsule and skin through a 26-gauge needle into the knee joint, the control and loosening groups were injected with 0.5 ml saline, and the infection groups were injected with 0.5 ml 10^5^ CFU *Staphylococcus aureus* (*S. aureus*) and 0.5 ml 10^8^ CFU *Staphylococcus epidermidis* (*S. epidermidis*), respectively; the bacterial concentration was determined as described above [10]). There were six rabbits for control group and ten in each of the other groups. Dressings were applied, and analgesia was administered for three days after surgery. For the control and loosening groups, penicillin was injected for three days postoperatively, while no prophylactic antibiotics were injected for the infection groups.

### PET/CT

PET/CT (uMI510, United Imaging Healthcare, Shanghai, China) was performed every two weeks using ^18^F-FDG and ^68^ Ga-FAPI in a randomly selected order on two consecutive days. ^18^F-FDG was synthesized according to the method described by Hamacher et al. [11] with modification through a computer-controlled apparatus. ^68^ Ga-FAPI (TanzhenBio Co., Ltd, Nanchang, China) was synthesized in house as previously reported [10]. Under sedation (a mixture solution of xylazine hydrochloride, chlorpromazine and sterile saline, 0.6 ml per rabbit), each rabbit was injected with approximately 37 MBq (1 mCi) and scanned one hour later. The PET acquisition time for ^18^F-FDG and ^68^ Ga-FAPI with one bed position was 5 min per scan. The attenuation was corrected using CT data, and the image was reconstructed using the standard ordered-subset expectation maximization algorithm. The dead time, decay, photon attenuation and the other effects were corrected for PET data.

Two junior nuclear medicine physicians blinded to the radiopharmaceuticals, the weeks of examination and the grouping were trained by a senior nuclear medicine physician to draw a 3D view of interest (VOI); they worked independently when performing this task. It was considered feasible that the results of the maximum standardized uptake value (SUVmax) were consistent. If the difference between the calculation of the mean SUV (SUVmean) and metabolic target volume (MTV) was less than 20%, the data were considered valid, and the final value was represented as the average of the two corresponding data [10].

### Serological examination

Blood samples were drawn from the central ear arteries, and sandwich ELISA (RayBiotech Life, Inc.) was used to detect C-reactive protein (CRP), interleukin-6 (IL-6) and FAP every two weeks after the operation according to the instructions.

### Micro-CT

Rabbits were euthanized by injecting an overdose of sodium pentobarbital intravenously. The part from the lower 1/2 of the femur to the upper 1/2 of the tibia was completely amputated through the aseptic technique. After wrapping with sterile preservative film, micro-CT (Quantum GX2, PerkinElmer, Waltham, Massachusetts, USA) was performed with 90 kV, 88 uA, FOV of 72 for four minutes. The bone surface area (BS)/bone volume (BV), BS/ trabecular volume (TV) and BV/TV were calculated for the targeted VOI.

### Tissue bacterial culture and pathology

After micro-CT, the soft tissue around the knee was dissected, partly for bacterial culture (where no bacterial growth for 72 h was defined as negative) and partly for pathological examination (which consisted of H&E and immunohistochemistry (IHC)), as previously reported [12].

### Pullout test and scanning electron microscopy (SEM)

**Fig. 1:**
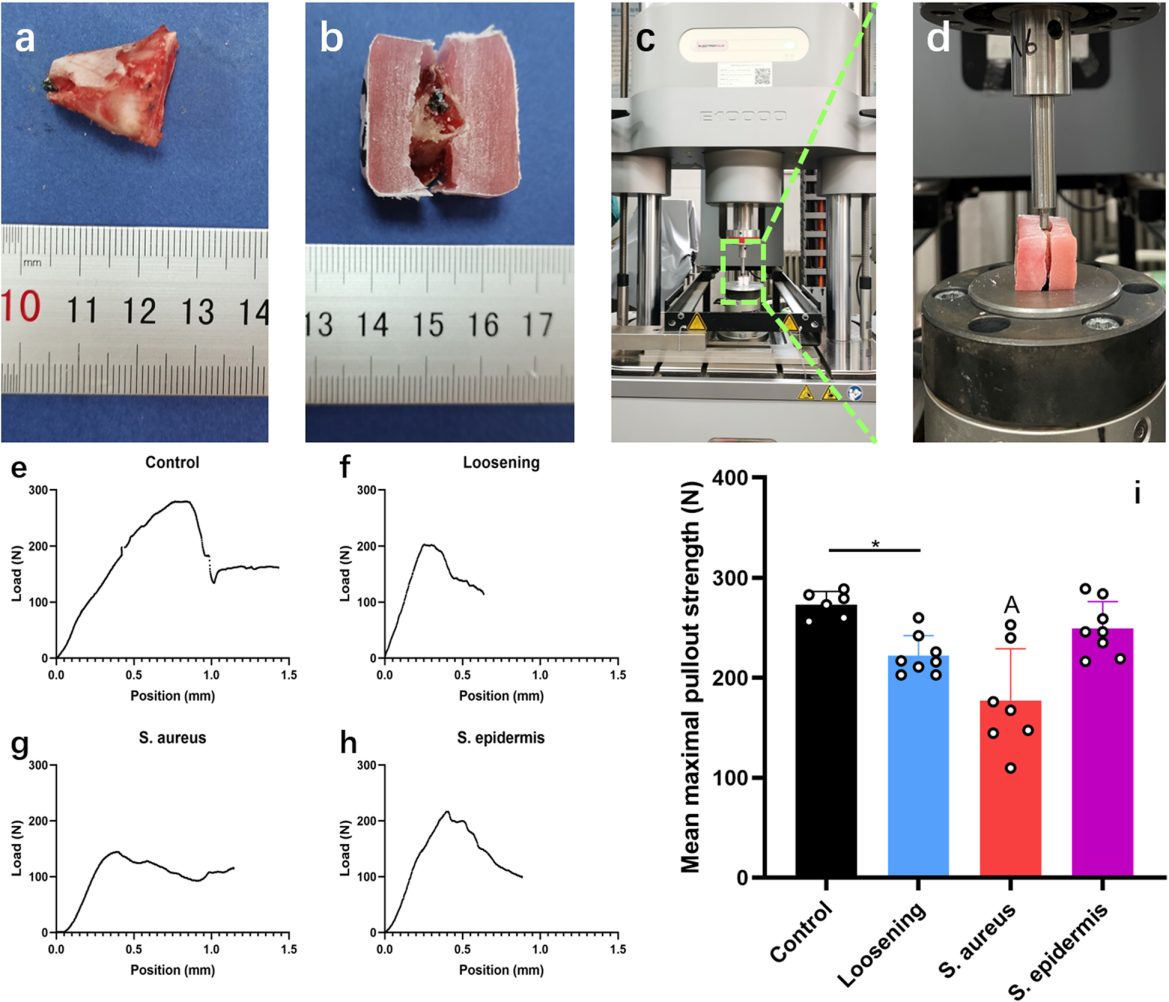
Pullout test. **a** Soft tissue was removed from the tibia. **b** The bone block was fastened by denture acrylic. **c** Distant view of the machine. **d** Close-up view of the objective table. **e** Representative image of the pullout strength of the control group. **f** Representative image of the pullout strength of the loosening group. **g** Representative image of the pullout strength of the *S. aureus* group. **h** Representative image of the pullout strength of the *S. epidermidis* group. **i** Mean maximal pullout strength of the four groups. *, *P* < 0.05; A, significantly different from other groups (*P* < 0.05)

Finally, the tibia containing the screw underwent a pullout test, and the screw from the femur underwent SEM. For the pullout test (Fig. 1a-d), the soft tissue around the tibia was removed as much as possible, and the distal end of the screw was exposed. Then, this bone block was fastened with denture acrylic. When the denture acrylic solidified, the shape of the bone block was cut to make the screw perpendicular to the ground when the bone block was placed on the objective table. The machine was started when the detector was adjusted to approximately 1 mm above the distal end of the screw at 5 Hz/s, and the maximum force value was recorded. For SEM, screws removed from the femur were fixed in 2.5% glutaric dialdehyde for 24 h, rinsed with distilled water three times and then dehydrated in a freeze drier. After the screw was mounted on an aluminium block stub and sputter-coated with gold–palladium, SEM was performed.

### Statistical analysis

Data are expressed as the mean ± standard deviation (SD). A paired t test was used for intra-group comparisons, and analysis of variance (ANOVA) was used for inter-group comparisons. Correlations between PET-derived metrics and other tests were analysed by Pearson’s rank correlation. All tests were 2-tailed, and a *p* value < 0.05 was considered statistically significant. GraphPad Prism 8.0.2 (GraphPad Software, San Diego, CA, USA) was used to perform statistical analyses.

## Results

The tissue cultures of the control group were all negative. One rabbit from the loosening group developed a femoral fracture during surgery (Supplementary Fig. 1), and one was tissue culture positive. One rabbit in the *S. aureus* group died after two weeks, and two died after four weeks; additionally, two rabbits in the *S. epidermidis* group did not develop infection. These seven rabbits were excluded from further analysis.

### Model validation

The pullout strength of the control group was 273.43 ± 12.96 N. In the loosening and *S. epidermidis* groups, the values were 222.24 ± 19.88 N and 249.31 ± 26.98 N, respectively. Finally, the pullout strength of the *S. aureus* group was 176.91 ± 52.09 N (Fig. 1e-i), which could be attributed to cortical bone destruction by *S. aureus*.

**Fig. 2:**
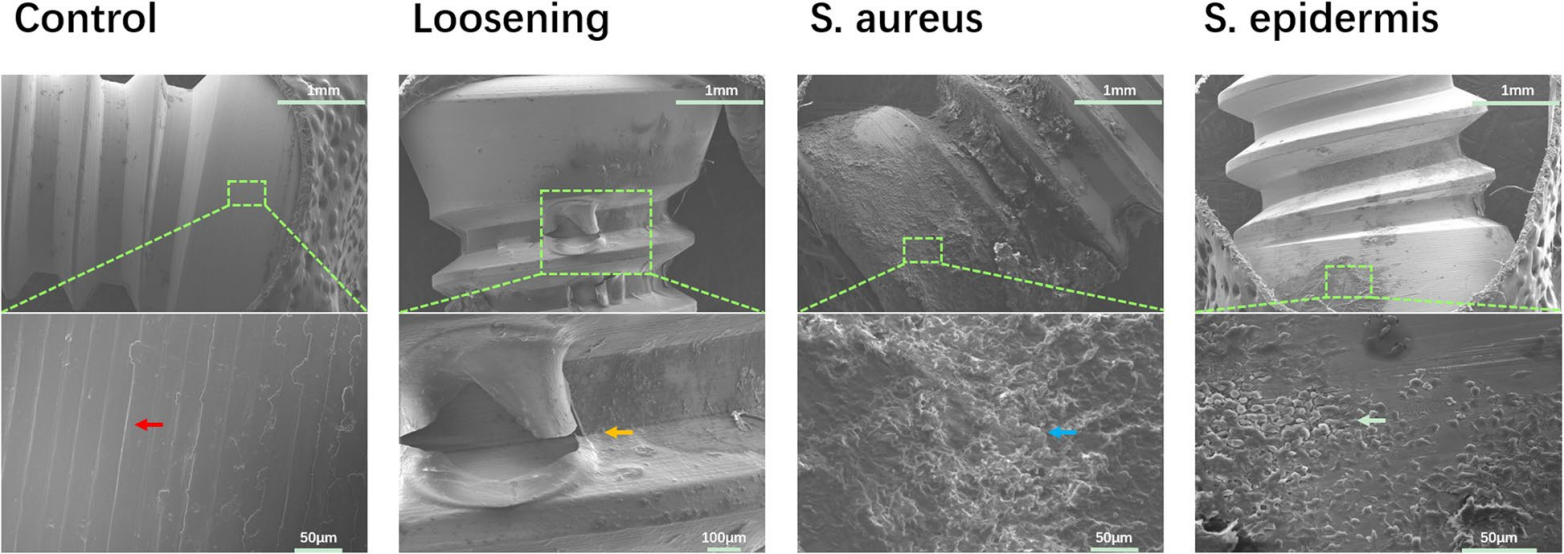
Scanning electron microscope images of the proximal end of the screw from the femur. Micrographs on the upper panels show the sections of entire screws. Micrographs in the lower panels represent magnifications of the area framed in the fluorescent green box. Images are representative of five replicates with similar results. Red arrow, thread of the screw. Brown arrow, ruptured fibrous membrane. Blue arrow, biofilm-like extracellular matrix. White arrow, sparse microflora

Screws from the femur were subjected to SEM (Fig. 2). In the control group, the thread of the screw was clearly visible. In the loosening group, a ruptured fibrous membrane could be observed. In the *S. aureus* group, a large number of bacteria and extensive biofilms were formed. In the *S. epidermidis* group, small numbers of bacteria and scarce biofilms could be detected. All of these results demonstrated the feasibility of this animal model.

### PET/CT examination

**Fig. 3:**
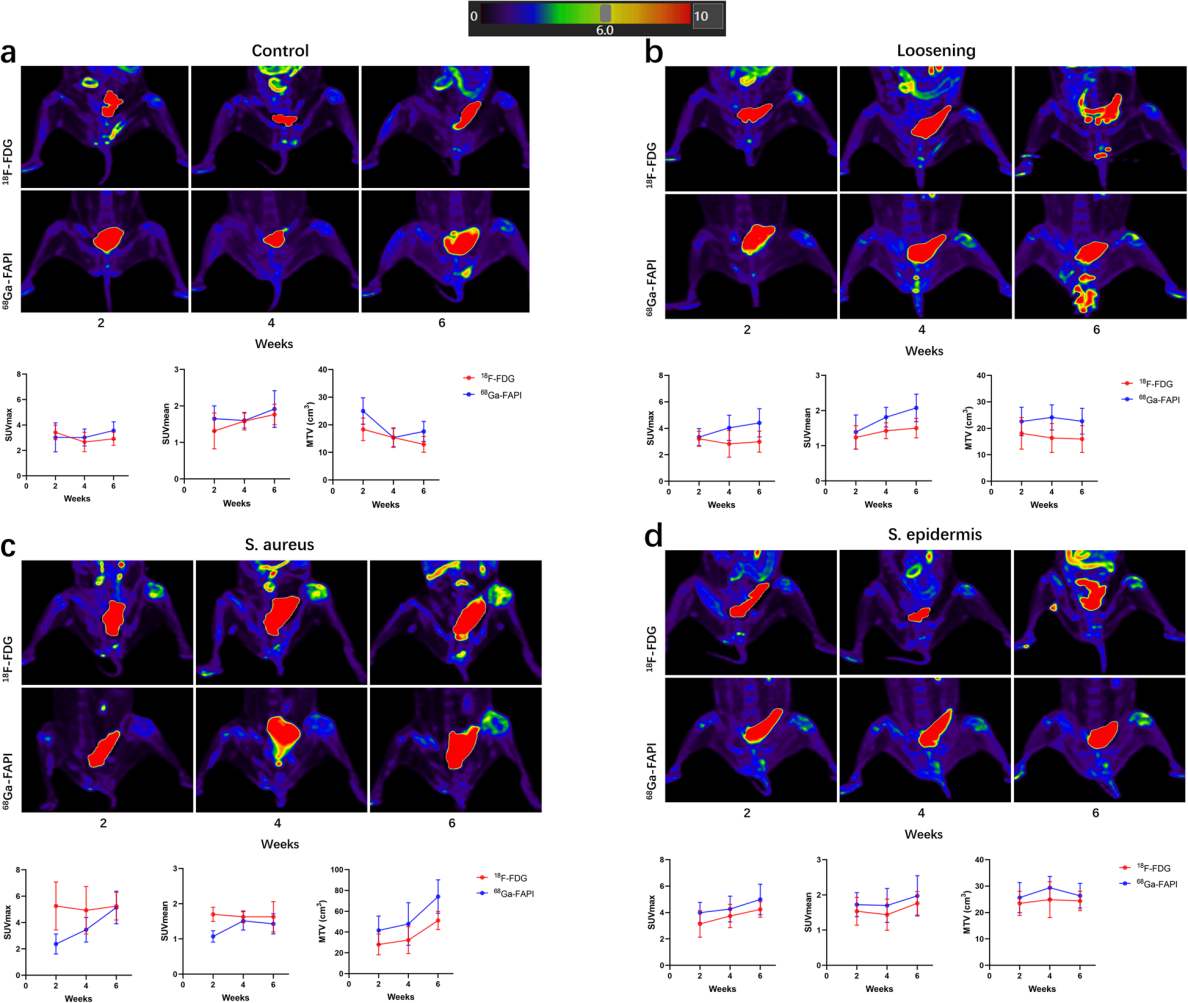
Representative images and results of SUVmax, SUVmean and MT(target)V of each group

In the control group, there was no significant uptake of ^18^F-FDG in the second week or in the fourth or sixth week. For ^68^ Ga-FAPI, the second week had the lowest SUVmax, which gradually increased in the following tests (Fig. 3a, Table 1). Since arthritis and osteophytes could express FAP and this surgery could also cause damage in the joint, it was assumed that degeneration occurred in this model.

**Table 1:** Original data of SUVmax, SUVmean and MT(target)V of each group

	Control		Loosening		S. aureus		S. epidermis	
	^18^F-FDG	^68^ Ga-FAPI	^18^F-FDG	^68^ Ga-FAPI	^18^F-FDG	^68^ Ga-FAPI	^18^F-FDG	^68^ Ga-FAPI
First week								
SUVmax	3.42 ± 0.57	3.03 ± 1.14	3.21 ± 0.57	3.34 ± 0.65	5.26 ± 1.82	2.37 ± 0.76	3.14 ± 1.01	3.99 ± 0.78
SUVmean	1.32 ± 0.49	1.65 ± 0.35	1.24 ± 0.33	1.39 ± 0.49	1.70 ± 0.20	1.07 ± 0.16	1.54 ± 0.40	1.73 ± 0.34
MTV	18.35 ± 4.06	24.97 ± 4.79	18.08 ± 5.97	22.59 ± 5.38	28.16 ± 9.98	45.66 ± 13.78	23.48 ± 4.58	25.66 ± 5.73
Fourth week								
SUVmax	2.67 ± 0.76	3.02 ± 0.66	2.83 ± 1.01	4.04 ± 0.95	4.93 ± 1.80	3.46 ± 0.93	3.74 ± 0.88	4.26 ± 0.98
SUVmean	1.58 ± 0.24	1.60 ± 0.21	1.43 ± 0.23	1.81 ± 0.28	1.63 ± 0.17	1.51 ± 0.26	1.44 ± 0.44	1.70 ± 0.48
MTV	15.38 ± 3.55	15.38 ± 3.34	16.35 ± 5.48	24.10 ± 4.75	32.30 ± 12.92	47.81 ± 20.48	24.88 ± 6.77	29.38 ± 4.30
Sixth week								
SUVmax	2.92 ± 0.52	3.57 ± 0.69	2.99 ± 0.80	4.41 ± 1.07	5.24 ± 1.05	5.14 ± 1.23	4.24 ± 0.59	4.99 ± 1.15
SUVmean	1.77 ± 0.28	1.92 ± 0.50	1.50 ± 0.28	2.08 ± 0.39	1.63 ± 0.43	1.43 ± 0.29	1.76 ± 0.33	1.96 ± 0.57
MTV	12.93 ± 2.89	17.58 ± 3.63	15.95 ± 5.06	22.69 ± 4.86	51.13 ± 8.65	74.13 ± 16.07	24.39 ± 3.64	26.34 ± 4.69

In the loosening group (Fig. 3b, Table 1), the manifestations of ^18^F-FDG were similar to those of the control group. For ^68^ Ga-FAPI, the mean SUVmax value was larger than that of the control group from the second week.

In the *S. aureus* group (Fig. 3c, Table 1), the SUVmax of ^18^F-FDG was persistently in a relatively high range, while the SUVmax of ^68^ Ga-FAPI showed a completely different performance. In the second week, the SUVmax of ^68^ Ga-FAPI was the lowest among the four groups (*P* < 0.05). As the number of weeks increased, so did the value of SUVmax, and in the sixth week, the mean value of ^68^ Ga-FAPI was highest among the four groups (no significant difference). No basic research has focused on this phenomenon, and our previous report made assumptions about it.

In the *S. epidermidis* group (Fig. 3d, Table 1), the SUVmax values of ^18^F-FDG and ^68^ Ga-FAPI were not statistically significant from those of the control group during the second week; thereafter, they gradually increased, and in the sixth week, those of the *S. epidermidis* group were significantly different from those of the control group (*P* < 0.05). Since the leucocyte chemotaxis of the *S. epidermidis* group was far weaker than that of the *S. aureus* group, it was foreseeable that ^18^F-FDG might not initially capture the disease well. For ^68^ Ga-FAPI, although there was no significant difference between the *S. epidermidis* and loosening groups in the second week, the average SUVmax of the *S. epidermidis* group was higher than that of the loosening group (4.0 ± 0.8 vs. 3.3 ± 0.6); in addition, the performance characteristics of the *S. epidermidis* group were different from those of the *S. aureus* group*,* and it was speculated that the low-virulence bacteria could increase the secretion of FAP to a certain degree.

For SUVmean, no clear conclusions could be drawn independently of SUVmax. Furthermore, in clinical cases, it was easy to draw VOIs around solid tumours but difficult to draw them around the hip prosthesis, which limited the use of SUVmean; the same was true of SUVpeak, total lesion glycolysis, and total lesion FAPI.

As far as MT(target)V is concerned, it also had the limitation mentioned above, but it should be noted that almost all MT(target)Vs of ^68^ Ga-FAPI were larger than those of ^18^F-FDG, which was in accord with our previous report, and this property might allow ^68^ Ga-FAPI to be more sensitive and to view a wider range of lesions.

### Pathological analysis

**Fig. 4:**
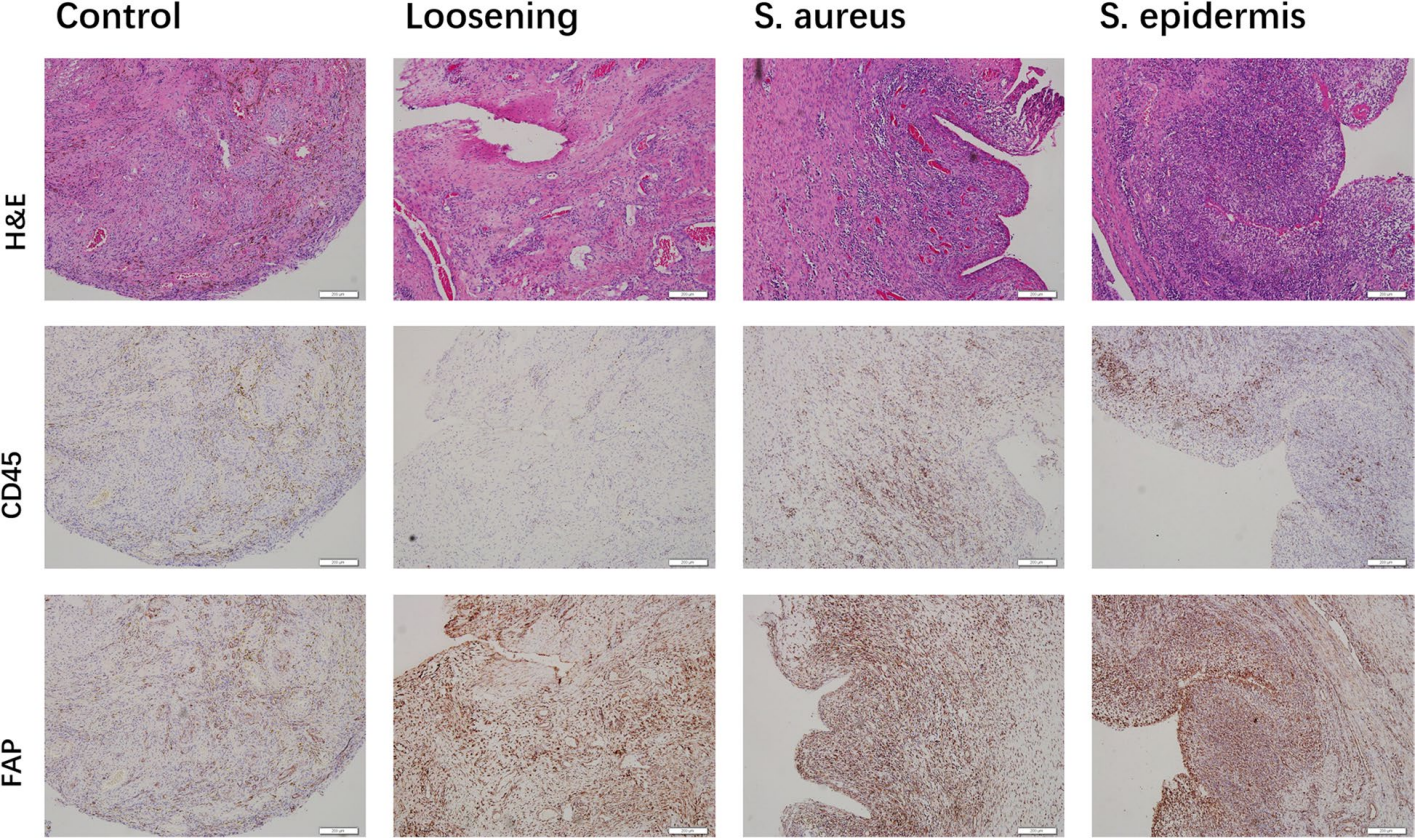
Analysis of models by pathology in parallel PET/CT scan in the sixth week. First row: representative H&E staining showing abundant leukocytes in the *S. aureus* and *S. epidermidis* groups and activated fibroblasts in the loosening group; second and third row: immunohistochemistry for CD45 and FAP. Images are representative of five biological replicates (scale = 200 µm)

Pathology was performed to validate the consistency of PET/CT in the sixth week (Fig. 4). H&E staining showed that abundant leukocytes were expressed in the *S. aureus* and *S. epidermidis* groups, and in the loosening group, abundant activated fibroblasts were detected. IHC included CD45 and FAP. Strikingly, CD45 was expressed in both infection groups. As for FAP, the loosening, *S. aureus* and *S. epidermidis* groups all had intense expression, and the control group also had moderate expression. The abovementioned results were consistent with the PET/CT results.

### Serological examination

**Fig. 5:**
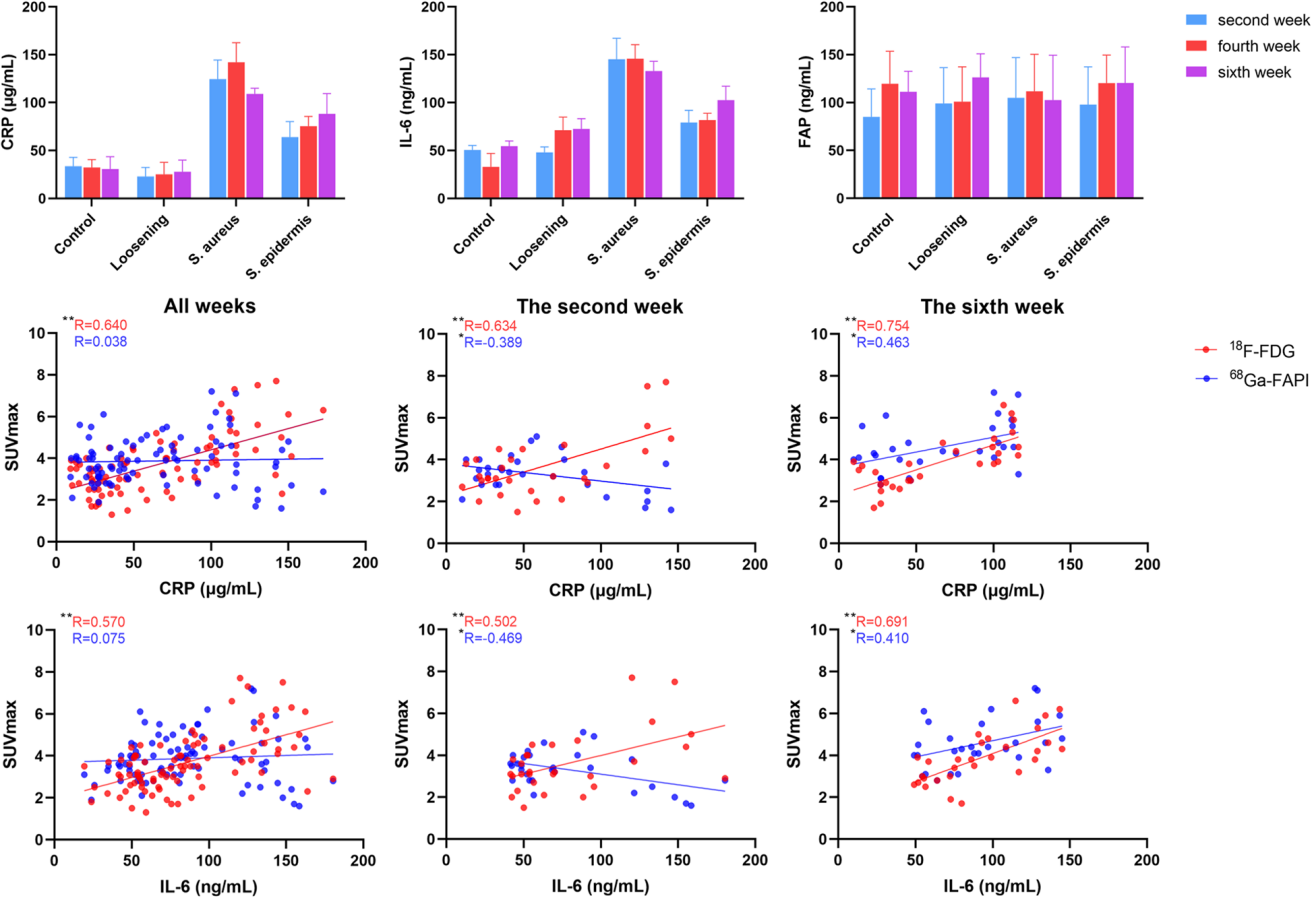
Serological examination and the linear correlation between SUVmax and serological results

Regarding the serological results (Fig. 5), the highest levels of CRP and IL-6 at every timepoint were found in the *S. aureus* group, followed by the *S. epidermidis* group. The CRP levels in the control and loosening groups were not significant; however, the IL-6 levels in the loosening group were significantly different from those in the control group in the fourth and sixth weeks. For FAP, no clear conclusions could be drawn in intra- or inter-group comparison, which was unexpected and yet somewhat reasonable, since several articles have reported that some diseases have FAP expression, but serum FAP remains unchanged.

Next, linear regressions between SUVmax and serological results were performed. For ^18^F-FDG, SUVmax was positively correlated with CRP and IL-6; however, 68 Ga-FAPI showed a completely different performance. In all weeks, there was no significant correlation between the SUVmax of ^68^ Ga-FAPI and CRP and IL-6, while SUVmax showed a negative and positive correlation with CRP and IL-6 in the second and sixth weeks, respectively. This phenomenon indicated that FAP was less expressed at the lesion site in the early stage of systemic inflammation and more expressed in the late stage, especially during infection. Whether this property of ^68^ Ga-FAPI led to a weaker role in acute infection or to a new therapeutic target for chronic infection deserves further study. For serum FAP, there was no significant correlation with SUVmax, ^18^F-FDG or ^68^ Ga-FAPI (data not shown).

### Micro-CT

**Fig. 6:**
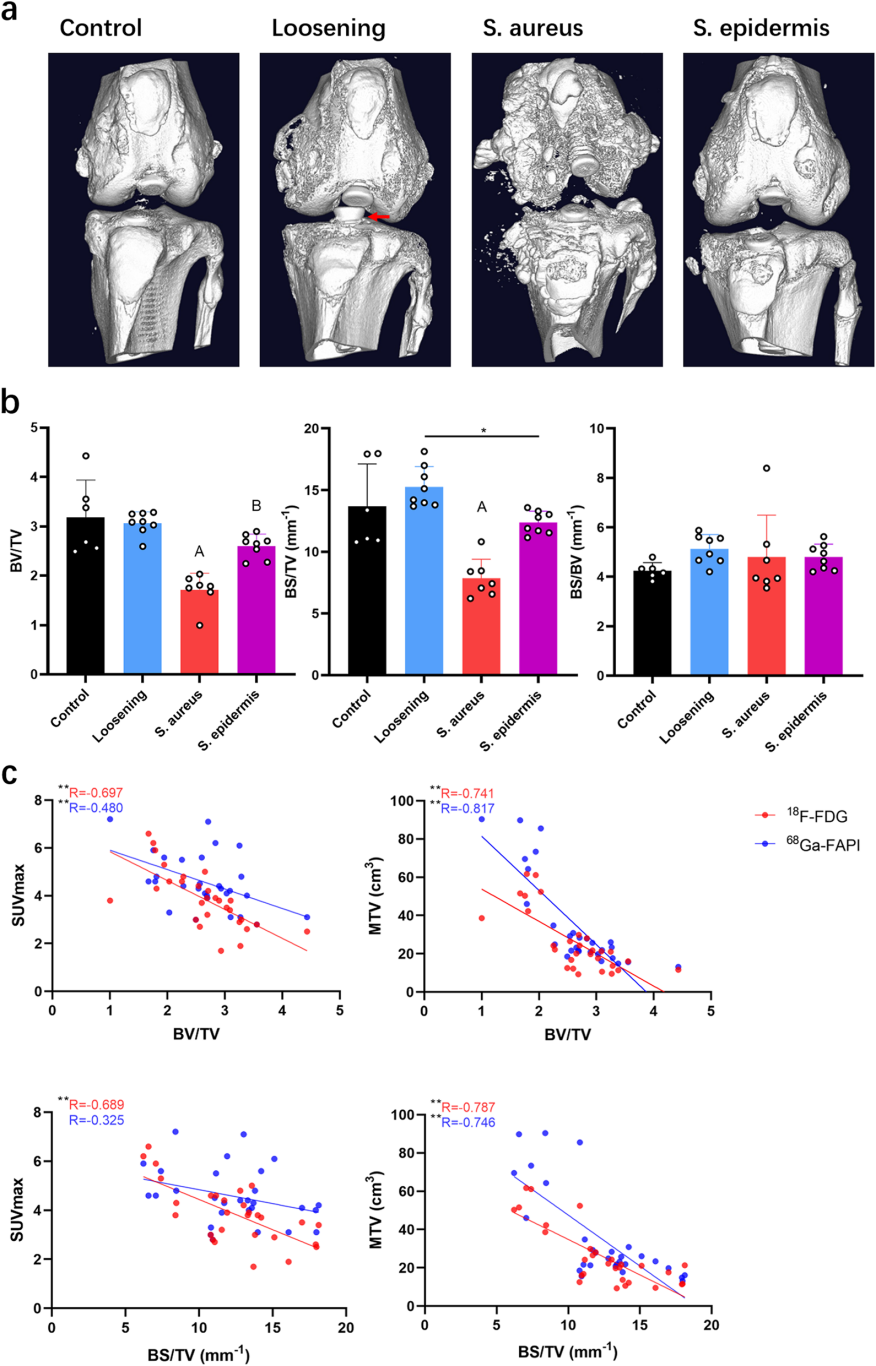
Micro-CT. a, Representative images. b, BV/TV, BS/TV and BS/BV results. *, *P* < 0.05; A, significantly different from the other groups (*P* < 0.05); B, significantly different from the control and loosening groups. Red arrow, retreat of the screw

Micro-CT was performed to observe the change in the bone (Fig. 6). It was apparent to the naked eye that the bone morphology of the *S. aureus* group was severely corroded, which was unsurprising, as *S. aureus* is far more erosive than *S. epidermidis*.

The BV/TV and BS/TV values of the *S. aureus* group were significantly different from those of other groups (*P* < 0.05); in the *S. epidermidis* group, the BV/TV value was significantly different from those of the control and loosening groups (*P* < 0.05), and the BS/TV value was significantly different from that of the loosening group (*P* < 0.05). The expression of FAP was thought to be associated with bone erosion, so linear regressions between SUVmax, SUVmean and MT(target)V and BV/TV and BS/TV were performed. Although both the SUVmax and the MT(target)V of ^18^F-FDG or ^68^ Ga-FAPI were negatively correlated with BV/TV and BS/TV, the MT(target)V of ^68^ Ga-FAPI was more sensitive than other indicators.

## Discussion

The aim of this study was to build a joint replacement loosening model based on the previous model and evaluate the performance characteristics of ^18^F-FDG and ^68^ Ga-FAPI in rabbit models of *S. aureus* infection, *S. epidermidis* infection, and aseptic loosening as well as a control group.

In this longitudinal observation, *S. aureus* and *S. epidermis* showed different uptake patterns in longitudinal observation. In the second week of ^68^ Ga-FAPI, the SUVmax and SUVmean of the *S. aureus* group were far less than those of the *S. epidermidis* group. In the next two tests, the values of the *S. aureus* group rose significantly and were equal to those of the *S. epidermidis* group. For the loosening group, a narrow range of high uptake was observed since the fourth week, which was believed to be caused by fibroblast activation resulting from the friction between the loose screw and the articular cavity. Although the SUVmax values in the loosening, *S. aureus* and *S. epidermidis* groups at the sixth week were nearly the same, their changes were markedly different over the course of the three tests. Meanwhile, the MT(target)V of the loosening group was larger than that of the control group, and uptake was visible along the loosening screw, which demonstrated that ^68^ Ga-FAPI has a certain application value in diagnosing prosthesis loosening. For ^18^F-FDG, although the SUVmax and SUVmean of the *S. aureus* group were significantly larger than those of the *S. epidermidis* group in all three tests, the discrimination of the control and loosening groups was blurry. In addition, almost all MT(target)Vs of ^68^ Ga-FAPI were larger than those of ^18^F-FDG. In summary, the mechanics of bacterial imaging of ^68^ Ga-FAPI were different from those of ^18^F-FDG, and ^68^ Ga-FAPI showed higher sensitivity in the detection of loosening. The present study demonstrated the feasibility of ^68^ Ga-FAPI in the assessment of symptoms affecting joint replacements.

The aetiology of loosening includes two major aspects, namely, abrasive particles and initial instability [8]. In the present study, we constructed a new loosening model based on the second mode. Several loosening models have already been established, including an air pouching model in mice and a debris-induced loosening model in murines and rabbits [13–18]. The original aim of the debris-induced loosening model was to simulate the situation in which bone cement, metal, etc., stimulate macrophages to produce absorptive stimulators leading to osteolysis. Clinically, we could also see widening of the medullary cavity in patients with loosening, and combined with the above, this was also a source of instability. This property was similar to initial instability, which was why we built this loosening model. In addition, the head of the screw received continuous force in the articular cavity, which was closer to the real clinical situation. Meanwhile, SEM was used to observe the surface of the screw, and ruptured fibrous membranes were found in the loosening group, which further proved the feasibility of this model.

For the test of mechanical stability, some scholars used the same force to record the different displacements of the screws [19], while others recorded the maximum force at which the screw detached from the bone [17, 18], which was the pullout strength. The latter method was used in this experiment. The *S. aureus* group had the lowest pullout strength of all groups, and micro-CT confirmed this result. The pullout strength of the control group was higher than the value observed by She et al., which may be due to the different examination sites and implants. The pullout strength of the loosening group was lower than the value in their report, which may mean that this model had the potential to be faster and more significant.

Although the application of ^68^ Ga-FAPI in PJI has not yet been reported, the advantages of ^68^ Ga-FAPI over ^18^F-FDG could be seen from this experiment and other reports [20–24]. First, ^68^ Ga-FAPI was more sensitive to disease detection. It can detect degeneration and loosening of the joint, as well as infections. On this topic, it is important to consider how infection and loosening can be distinguished from one another. Although the SUVmax values of these groups were similar, the time courses of their variation were different, along with those of MT(target)V, which suggests that loosening and infection may cause different uptake patterns in the clinic; indeed, we found different uptake patterns in loosening and infection in clinical cases (Supplementary Fig. 2). In addition, with the development of PET/CT radiomics [25, 26], there is great interest in whether this method could be used to diagnose the types of bacteria responsible for the infection. Second, ^68^ Ga-FAPI did not have nonspecific muscle and intestinal uptake compared with ^68^ Ga-FAPI, which could increase the specificity of ^68^ Ga-FAPI. Third, ^68^ Ga-FAPI does not require fasting and shows a broader range of lesions with clearer boundaries, and the waiting time for examination may be shorter.

There were several limitations in this study. Due to the limited rabbit number, pathology of the second and fourth weeks was not performed. Then, many phenomena found in this experiment need to be studied in basic experiments. Finally, this study was performed only in animals, and clinical studies are urgently needed to verify the feasibility of ^68^ Ga-FAPI.

## Conclusion

In this study, SEM and pullout tests were used to validate the feasibility of a new loosening model. In the longitudinal observation of the control, loosening, *S. aureus* and *S. epidermidis* groups, ^68^ Ga-FAPI showed greater sensitivity and specificity than ^18^F-FDG in detecting diseases. In addition, almost all MT(target)Vs of ^68^ Ga-FAPI were larger than those of ^18^F-FDG, which meant that ^68^ Ga-FAPI had the potential to define the scope of lesions more accurately. Taken together, these results suggest that ^68^ Ga-FAPI has great potential in the diagnosis of PJI and has distinct advantages over ^18^F-FDG. However, the diagnostic efficacy of SUVmax was not satisfactory in ^68^ Ga-FAPI, and further study of the diagnostic criteria and basic research about the mechanism of FAP in infection and the expression site of FAP in infection is warranted.

## Supplementary Information

**Additional file 1. **

**Additional file 2. **

## Data Availability

All data generated or analysed during this study are included in this article and its supplementary information files. This experiment was approved by the Laboratory Animal Center of Chinese People’s Liberation Army General Hospital (2020-X16-93) and performed in accordance with the ARRIVE guidelines.
