# Supplementary figures and images for: 18F-FDG and 68 Ga-FAPI PET/CT for the evaluation of periprosthetic joint infection and aseptic loosening in rabbit models
Source: BMC Musculoskelet Disord. 2022 Jun 20;23:592. doi: 10.1186/s12891-022-05537-w (PMC9208226; doi:10.1186/s12891-022-05537-w)

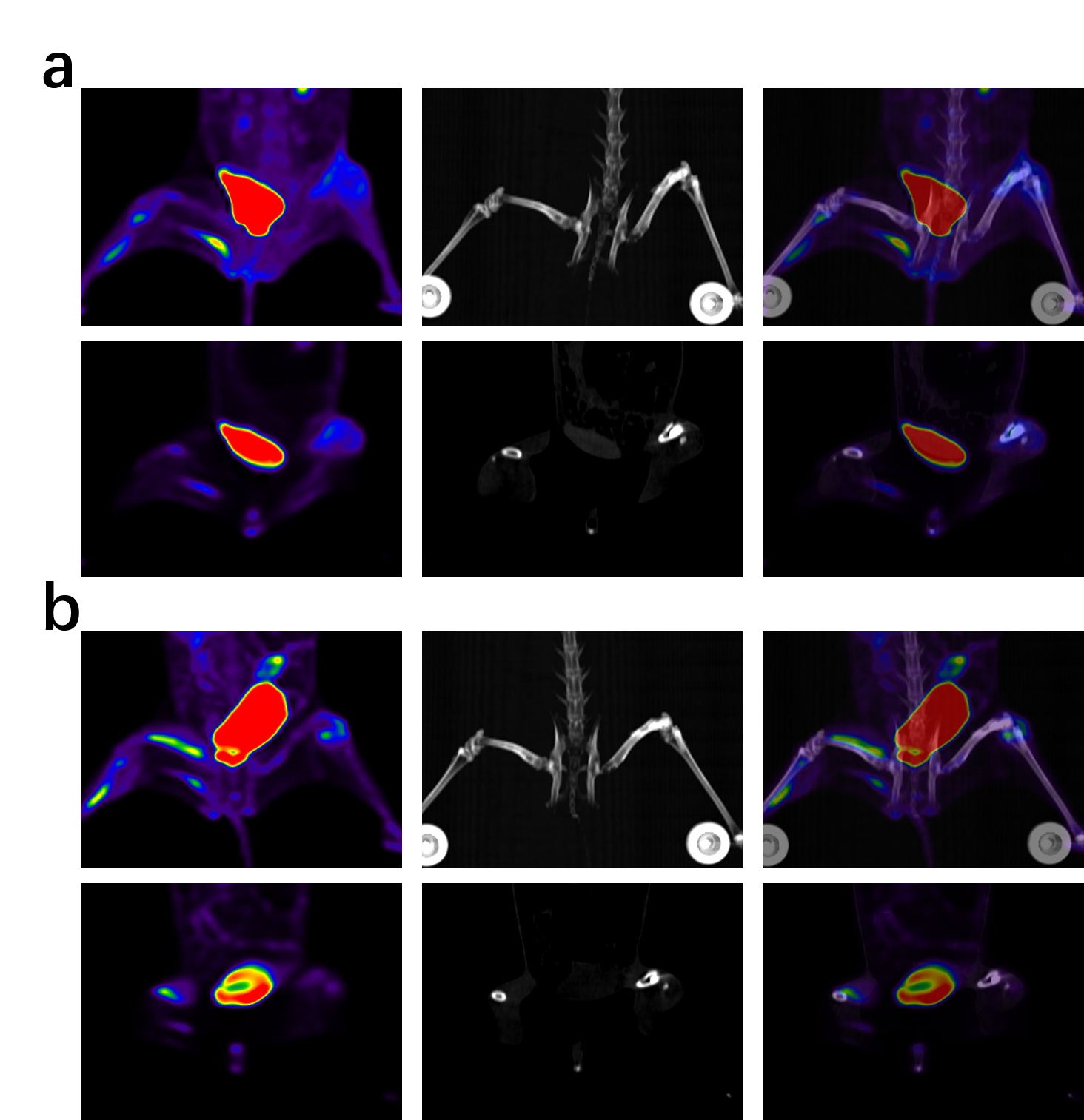

Supplement: Supplementary file 1 — Additional file 1. [file 12891_2022_5537_MOESM1_ESM.png]

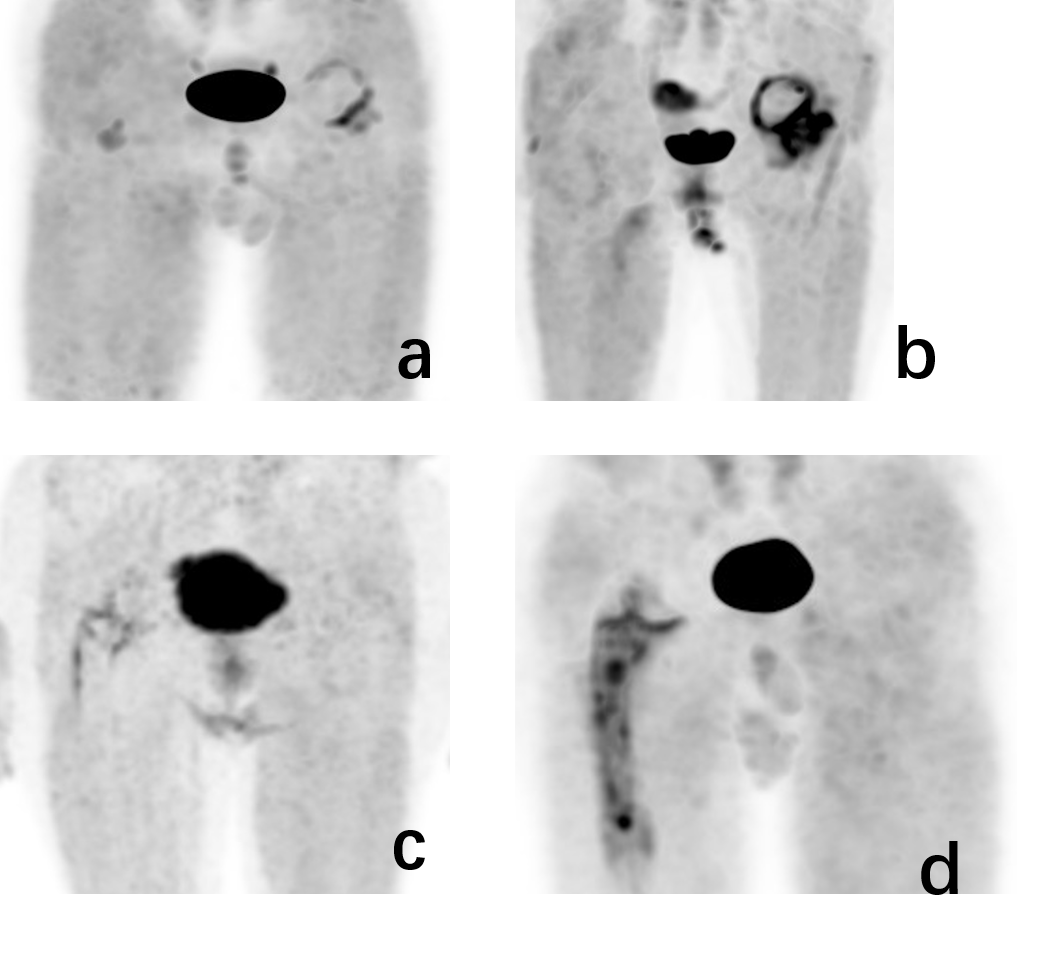

Supplement: Supplementary file 2 — Additional file 2. [file 12891_2022_5537_MOESM2_ESM.png]
